# Comparative effectiveness of antiviral treatment on household transmission of SARS-CoV-2: a retrospective cohort study using administrative data

**DOI:** 10.1186/s12879-025-11651-6

**Published:** 2025-09-29

**Authors:** Kazuhiko Ikeuchi, Makoto Saito, Kazuya Okushin, Yuki Arisato, Toshiyuki Kishida, Shinya Matsumoto, Akira Kado, Hiroshi Yotsuyanagi, Takeya Tsutsumi

**Affiliations:** 1 Department of Infectious Diseases, Graduate School of Medicine, The University of Tokyo, 7-3-1 Hongo, Bunkyo-ku, Tokyo, 113-8655 Japan; 2 Centre for Tropical Medicine and Global Health, Nuffield Department of Medicine, University of Oxford, Oxford, OX3 7LF UK; 3 Department of Infection Control and Prevention, Graduate School of Medicine, The University of Tokyo, 7-3-1 Hongo, Bunkyo-ku, Tokyo, 113-8655 Japan; 4 Department of Gastroenterology, Graduate School of Medicine, The University of Tokyo, 7-3-1 Hongo, Bunkyo-ku, Tokyo, 113-8655 Japan; 5 Division for Health Service Promotion, The University of Tokyo, 7-3-1 Hongo, Bunkyo-ku, Tokyo, 113-8655 Japan; 6 Department of Infectious Diseases and Applied Immunology, IMSUT Hospital of The Institute of Medical Science, The University of Tokyo, 4-6-1 Shirokanedai, Minato-ku, Tokyo, 108-8639 Japan

## Abstract

**Background:**

Antiviral treatment reduces influenza transmission and differs in effectiveness among agents. Although SARS-CoV-2 antivirals lower viral shedding, their role in preventing secondary household transmission and the differences between agents remain unclear.

**Methods:**

We conducted a retrospective cohort study using the JMDC administrative claims database in Japan. The study included married-couple households between 1 April and 31 August 2023, when the Omicron XBB variant was predominant. Households in which at least one person had been diagnosed with Coronavirus Disease 2019 (COVID-19) were included. We excluded households if the index patient did not receive antiviral treatment on day 0, or the spouse was diagnosed on day 0 or 1. The primary outcome was subsequent infection in the spouse by day 7. Cox proportional hazards models were used to estimate hazard ratios (HRs), after adjusting for potential confounders.

**Results:**

Of the 326,827 married-couple households, 5,398 met the inclusion criteria. Among them, 1,143 households (21.2%) experienced presumed secondary transmission by day 7. The cumulative transmission rate, estimated using the Kaplan–Meier method, was lower among hospitalized patients (*n* = 73, 11.0%, 95% confidence interval [CI]: 5.7–20.8%) than among outpatients (*n* = 5,325, 21.5%, 95% CI: 20.4–22.6%, *p* = 0.035). Transmission rates did not significantly differ among the outpatient antiviral groups: molnupiravir (*n* = 3,093, 21.3%, 95% CI: 19.9–22.8%), ensitrelvir (*n* = 1,907, 21.6%, 95% CI: 19.8–23.6%), and nirmatrelvir/ritonavir (*n* = 323, 22.8%, 95% CI: 18.6–27.8%, *p* = 0.74). In multivariable Cox analysis, male sex (adjusted HR 1.43, 95% CI: 1.26–1.63; *p* < 0.001), history of COVID-19 in the index patient (adjusted HR 0.50, 95% CI: 0.33–0.76; *p* = 0.001), and history of COVID-19 in the partner (adjusted HR 0.31, 95% CI: 0.21–0.45; *p* < 0.001) were significantly associated with transmission risk. Hospitalization tended to be associated with a lower risk of transmission (adjusted HR, 0.51; 95% CI, 0.25–1.03; *p* = 0.062).

**Conclusions:**

Household transmission rates were not statistically different among three different outpatient oral antiviral agents. Hospitalization was associated with a trend toward lower transmission rates, possibly due to physical isolation.

**Supplementary Information:**

The online version contains supplementary material available at 10.1186/s12879-025-11651-6.

## Background

SARS-CoV-2 is a single-stranded RNA virus that is primarily transmitted through short-range aerosols in person-to-person contact [1, 2]. Airborne transmission can occur, particularly in poorly ventilated environments [3]. Close contact with infected individuals plays a critical role in viral transmission, and household secondary transmission rates have been reported to be as high as around 30–50% during the Omicron era [4–7]. While infection control measures such as hand washing and adequate ventilation are effective in preventing SARS-CoV-2 infection, household transmission remains one of the most challenging settings to control.

Several antiviral agents are available for treating SARS-CoV-2 infections. Nirmatrelvir/ritonavir has been shown to substantially reduce the risk of hospitalization and death in high-risk outpatients [8]. Molnupiravir was shown to be less effective [9, 10]. Ensitrelvir, another 3CL protease inhibitor similar to nirmatrelvir, has been shown to accelerate symptom resolution in studies conducted during the Omicron period and has been approved for use in mild cases within 72 h of symptom onset in Japan [11]. Remdesivir, administered intravenously, is primarily used for hospitalized patients with severe Coronavirus Disease 2019 (COVID-19) but may also be administered to high-risk outpatients [12]. According to Japanese clinical guidance, oral antivirals can be prescribed to symptomatic COVID-19 patients who do not require supplemental oxygen. The decision to initiate treatment is made by the physician [13]. Among these agents, ensitrelvir can also be prescribed to patients without any known risk factors for severe disease.

Although antiviral agents reduce the viral load of SARS-CoV-2, it remains unclear whether they decrease transmission to others. In the case of influenza, antiviral treatment has been shown to reduce household transmission [14]. Furthermore, baloxavir achieves greater viral load reduction than oseltamivir and may be more effective in preventing transmission to household contacts [15]. An animal study using a ferret model, demonstrated that molnupiravir strongly suppressed transmission of SARS-CoV-2, while nirmatrelvir/ritonavir showed only partial suppression [16]. In that study, naïve ferrets were co-housed with SARS-CoV-2-infected ferrets 42 h after the infected animals had received antiviral treatment, which is quite different from the real-world household situations. Considering that patients with COVID-19 are known to shed the virus even before symptom onset [17], making it further uncertain whether treating infected individuals with antivirals after the onset of symptoms can effectively reduce subsequent household transmission.

We analyzed a large administrative claims database with family relationship information to assess whether the effects of different antiviral agents on household transmission varied by the agent.

## Methods

### Study design and description of the data source

We conducted a retrospective cohort study using data from the JMDC database, an administrative claims database that covers approximately 11 million individuals in Japan. The database includes insurance claims data, such as International Classification of Diseases, 10th Revision (ICD-10) diagnostic codes, treatments, and procedures, and family relationship information. Household relationships, including spouses, are explicitly recorded based on insurance enrollment data. Each insured employee is linked to their dependents (e.g., spouse, children) via a unique household identifier. Vaccination data were not available because Japan’s insurance system did not cover COVID-19 vaccination. As the database is derived from employer-based insurance, it primarily includes a relatively younger population. In Japan, individuals aged 75 years or older are covered by a separate insurance system; therefore, data for patients aged 75 years and older are unavailable.

The investigators had full access to the anonymized JMDC database, including diagnostic codes, prescription records, and family relationship data for all insured individuals during the study period. Data cleaning included the exclusion of households with incomplete or contradictory family relationship information, and cases with missing diagnosis dates or overlapping antiviral prescriptions.

### Study population

To avoid the influence of child-to-parent transmission, we restricted the study population to households consisting of married couples only. Households with missing spousal relationship data were excluded from the analysis. The viral load and transmission rates of SARS-CoV-2 may vary depending on the viral variant; therefore, we limited the study period to 1 April 2023, to 31 August 2023, when the Omicron XBB variant predominated [18] and all four currently available antiviral drugs against SARS-CoV-2 became available in Japan. We included households in which at least one person was diagnosed with COVID-19 during this period.

The index date (day 0) was defined as the earliest COVID-19 diagnosis between the two household members. We included only the first diagnosed case per household during the study period, and each individual was counted only once as either the index case or their partner. Households were excluded if either person was hospitalized before day 0. We also excluded households in which the index patient did not receive antiviral treatment (i.e., nirmatrelvir/ritonavir, ensitrelvir, molnupiravir, or remdesivir) on day 0, as untreated patients may differ substantially from those treated in terms of symptom onset, vaccination status, and underlying health conditions (i.e., bias by indication). In addition, couples in which both were diagnosed on the same day (day 0) or the following day (day 1) were excluded, as infections diagnosed in spouses on day 1 had likely already occurred prior to antiviral initiation in the index case, considering the typical incubation period of SARS-CoV-2 and the time lag between symptom onset and diagnosis [19]. Patients who received two or more antiviral agents on the same day were also excluded.

Hospitalizations of the index case or the partner between days 1 and 7 were not excluded from the main analysis, as exposures during this period could have occurred prior to hospitalization and still be relevant for transmission. As a sensitivity analysis, we also conducted an analysis excluding households in which the index patient was hospitalized between days 1 and 7 for any reasons, or the partner was hospitalized for non-COVID-19 reasons during the same period. Partners who were hospitalized for COVID-19 were analyzed as having the outcome (COVID-19 infection) in either case.

### Definitions and data Preparation

We collected data on the following variables at day 0: age, sex, prior COVID-19 infection (before 1 April 2023), and the presence of the following conditions in either spouse: solid tumors, leukemia, lymphoma, multiple myeloma, hematopoietic stem cell transplantation (HSCT), solid organ transplantation (SOT), heart failure, liver cirrhosis, dialysis, diabetes mellitus, connective tissue diseases, asthma, chronic obstructive pulmonary disease (COPD), interstitial lung disease, and oxygen use on the date of diagnosis as an indicator of disease severity. Diagnoses were based on the ICD-10 codes used as confirmed diagnoses (Supplementary Table 1) [20, 21]. The prescriptions of disease-specific medications were used in combination with the diagnostic names to ascertain the diagnoses of heart failure, diabetes, and asthma.

We defined the exposure windows for medications as follows: prescriptions of heart failure medications, diabetes medications, asthma inhalers, corticosteroids, and prophylaxis for *Pneumocystis jirovecii* pneumonia (PJP) within 90 days before diagnosis were considered active use; antineoplastic agents within 30 days were considered active; and anti-CD20 monoclonal antibodies within 365 days were considered active because of their prolonged biological effect [22].

### Statistical analysis

When a spouse of the index case was diagnosed with COVID-19, the date of symptom onset was defined as 0.5 days before the diagnosis date. We assessed whether household transmission occurred within 7 days (between day 2 and day 7) [7]. The patient characteristics were compared between households with and without presumed secondary transmission. Categorical variables were compared using the chi-square or Fisher’s exact tests, and continuous variables were compared using the Mann–Whitney U test.

Kaplan–Meier curves were used to visualize the time to secondary infection, and log-rank tests were used to compare the different antiviral treatments. Cox proportional hazards models were used to calculate the adjusted hazard ratios (HRs) for secondary household transmission after different antiviral treatments, adjusting for potential confounders including antiviral treatment group, age, sex, prior COVID-19 infection, hospitalization, immunocompromised status of the index patient, prior COVID-19 infection of the partner, and other variables found to be significant in univariable analyses. Age was included in the model as a categorical variable divided into quartile ranges: <49, 49–55, 56–60, and ≥ 61 years. Immunocompromised status was included based on the hypothesis that impaired immunity may lead to prolonged viral shedding and increased transmission risk [23]. Immunocompromised status included conditions such as solid tumors, leukemia, lymphoma, multiple myeloma, use of systemic corticosteroids, and prophylaxis for PJP. Variables with small sample sizes (e.g., anti-CD20 monoclonal antibody use) were excluded from the multivariable models. The proportional hazards assumption was evaluated by inspecting Nelson-Aalen estimator (cumulative hazard function). Variables that showed evidence of violating this assumption (such as multiple myeloma or dialysis in index patients) were excluded from the multivariable Cox model.

All statistical analyses were performed using Stata version 18 (StataCorp, College Station, TX, USA).

### Ethics

The Research Ethics Committee of the University of Tokyo approved this study (approval number 2024216NIe). The Ethics committee of University of Tokyo waived the requirement for informed consent because the study used fully anonymized administrative data that were not individually identifiable. Data confidentiality was strictly maintained in accordance with the Declaration of Helsinki and this study was reported following the Reporting of Studies Conducted Using Observational Routinely Collected Health Data (RECORD) statement [24].

## Results

### Patient selection

**Fig. 1:**
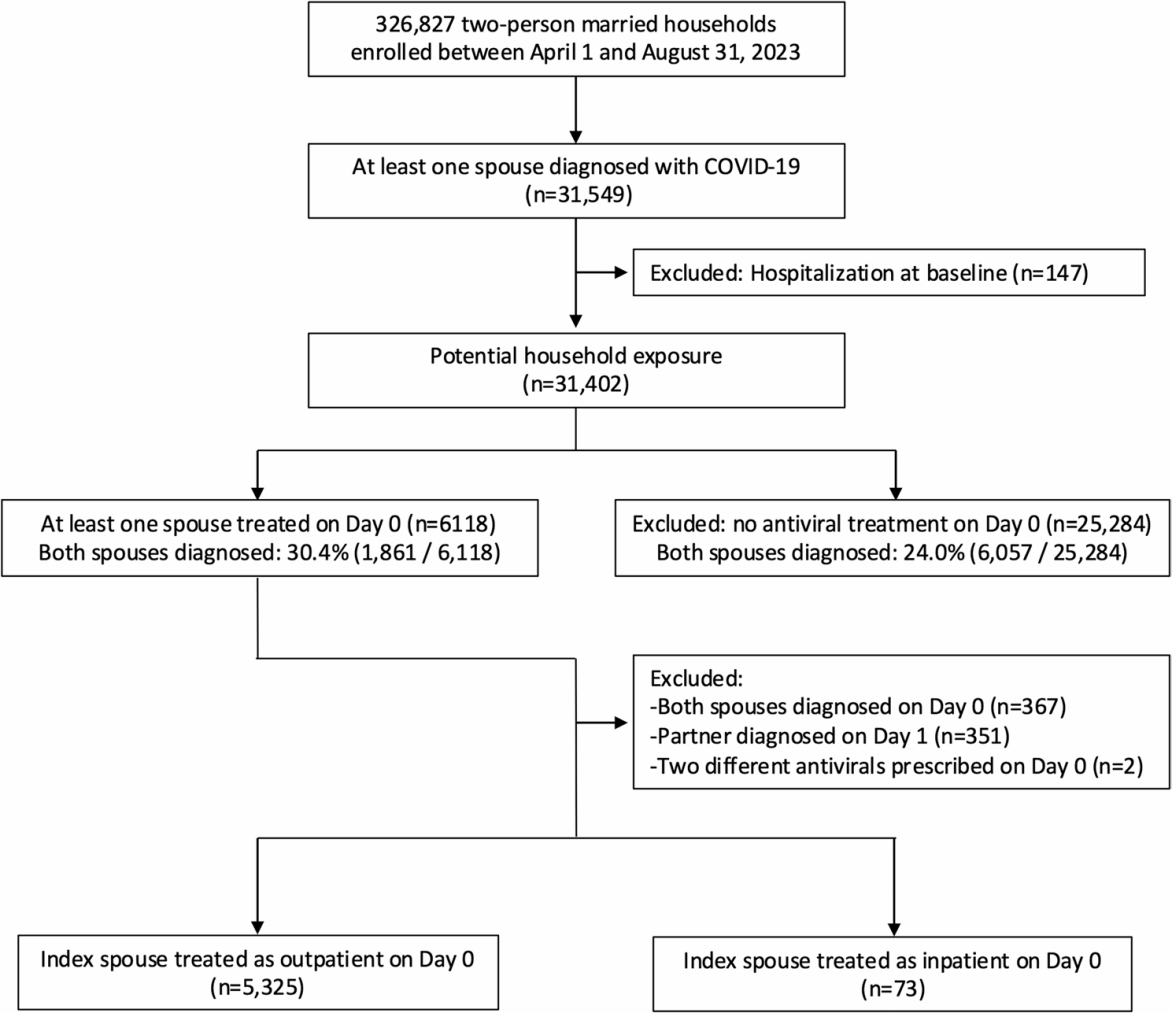
Study flow chart. Abbreviations: COVID-19

Between April 1 and August 31, 2023, a total of 326,827 married-couple households (653,654 individuals) were included in the database (Fig. 1). During this period, at least one person in 31,549 households was diagnosed with COVID-19. Among them, 147 households were excluded because either spouse had been hospitalized before day 0. Of the remaining households, 6,118 had at least one spouse who initiated antiviral treatment on day 0, whereas 25,384 households, neither spouse received antiviral treatment on that day. Between day 0 and day 7, both spouses were diagnosed with COVID-19 in 30.4% (1,861/6,118) of the treated households and in 24.0% (6,057/25,284) of the untreated households.

Among the 6,118 treated households, 367 were excluded because both spouses were diagnosed on day 0, and 351 were excluded because the second spouse was diagnosed on day 1 and two were excluded due to simultaneous prescription of two different antiviral agents. After these exclusions, 5,398 households were included in the final analysis. Among them, 5,325 patients initiated antiviral treatment in an outpatient setting, and 73 patients were hospitalized and began antiviral therapy on the day of diagnosis.

### Patient and partner’s characteristics

The characteristics of the index patients and their partners are shown in Table 1. Among the index patients, 63.8% (3,444/5,398) were male. The median age of the index patients was 56.0 years (interquartile range [IQR], 49.0–61.0), and the median age of their partners was 55.0 years (IQR, 48.0–60.0). Regarding outpatient antiviral treatment, 3,093 patients (57.3%, 3,093/5,398) received molnupiravir, 1,907 (35.3%, 1,907/5,398) received ensitrelvir, 323 (6.0%, 323/5,398) received nirmatrelvir/ritonavir, and two received remdesivir (0.0%, 2/5,398). A total of 73 patients (1.4%, 73/5,398) were hospitalized and received antiviral treatment on day 0, with remdesivir being the most commonly used agent (*n* = 53).

**Table 1:** Characteristics of index patients and partners

	Total	Household transmission		
		no	yes	
	*N* = 5,398	*N* = 4,255	*N* = 1,143	*P* value
Index patient				
Age	56.0 (49.0–61.0)	56.0 (49.0–61.0)	56.0 (48.0–60.0)	0.41
Male	3,444 (63.8%)	2,633 (61.9%)	811 (71.0%)	< 0.001
Outpatient treatment	5,325 (98.6%)	4,190 (98.5%)	1,135 (99.3%)	0.031
Molnupiravir	3,093 (57.3%)	2,440 (57.3%)	653 (57.1%)	0.90
Ensitrelvir	1,907 (35.3%)	1,498 (35.2%)	409 (35.8%)	0.72
Nirmatrelvir/ritonavir	323 (6.0%)	250 (5.9%)	73 (6.4%)	0.52
Remdesivir	2 (0.0%)	2 (0.0%)	0 (0.0%)	0.46
Inpatient treatment	73 (1.4%)	65 (1.5%)	8 (0.7%)	0.031
Molnupiravir	16 (0.3%)	15 (0.4%)	1 (0.1%)	0.22
Ensitrelvir	1 (0.0%)	1 (0.0%)	0 (0.0%)	1.00
Nirmatrelvir/ritonavir	3 (0.1%)	3 (0.1%)	0 (0.0%)	1.00
Remdesivir	53 (1.0%)	46 (1.1%)	7 (0.6%)	0.15
Oxygen use on day 0	24 (0.4%)	23 (0.5%)	1 (0.1%)	0.043
History of COVID-19	253 (4.7%)	230 (5.4%)	23 (2.0%)	< 0.001
Solid tumor	544 (10.1%)	427 (10.0%)	117 (10.2%)	0.84
Leukemia	13 (0.2%)	12 (0.3%)	1 (0.1%)	0.32
Lymphoma	39 (0.7%)	33 (0.8%)	6 (0.5%)	0.37
Multiple myeloma	14 (0.3%)	11 (0.3%)	3 (0.3%)	1.00
Heart failure	391 (7.2%)	302 (7.1%)	89 (7.8%)	0.42
Asthma	252 (4.7%)	193 (4.5%)	59 (5.2%)	0.37
COPD	84 (1.6%)	65 (1.5%)	19 (1.7%)	0.74
Interstitial lung disease	98 (1.8%)	73 (1.7%)	25 (2.2%)	0.29
Liver cirrhosis	17 (0.3%)	16 (0.4%)	1 (0.1%)	0.15
Dialysis	53 (1.0%)	44 (1.0%)	9 (0.8%)	0.45
Diabetes mellitus	712 (13.2%)	546 (12.8%)	166 (14.5%)	0.13
Collagen disease	326 (6.0%)	255 (6.0%)	71 (6.2%)	0.78
HSCT	1 (0.0%)	1 (0.0%)	0 (0.0%)	1.00
SOT	15 (0.3%)	12 (0.3%)	3 (0.3%)	1.00
Corticosteroid use	305 (5.7%)	249 (5.9%)	56 (4.9%)	0.35
Antineoplastic agents	6 (0.1%)	6 (0.1%)	0 (0.0%)	0.20
Anti-CD20 therapy	6 (0.1%)	6 (0.1%)	0 (0.0%)	0.35
Prophylaxis for PJP	44 (0.8%)	37 (0.9%)	7 (0.6%)	0.39
Partners				
Age	55.0 (48.0–60.0)	55.0 (48.0–60.0)	55.0 (47.0–60.0)	0.24
Male	1,954 (36.2%)	1,622 (38.1%)	332 (29.0%)	< 0.001
History of COVID-19	426 (7.9%)	398 (9.4%)	28 (2.4%)	< 0.001
Solid tumor	448 (8.3%)	358 (8.4%)	90 (7.9%)	0.56
Leukemia	10 (0.2%)	8 (0.2%)	2 (0.2%)	1.00
Lymphoma	25 (0.5%)	20 (0.5%)	5 (0.4%)	1.00
Multiple myeloma	3 (0.1%)	2 (0.0%)	1 (0.1%)	0.51
Heart failure	223 (4.1%)	174 (4.1%)	49 (4.3%)	0.77
Asthma	216 (4.0%)	175 (4.1%)	41 (3.6%)	0.42
COPD	40 (0.7%)	35 (0.8%)	5 (0.4%)	0.24
Interstitial lung disease	72 (1.3%)	53 (1.2%)	19 (1.7%)	0.28
Liver cirrhosis	19 (0.4%)	15 (0.4%)	4 (0.3%)	1.00
Dialysis	10 (0.2%)	7 (0.2%)	3 (0.3%)	0.45
Diabetes mellitus	422 (7.8%)	329 (7.7%)	93 (8.1%)	0.65
Collagen disease	301 (5.6%)	230 (5.4%)	71 (6.2%)	0.29
HSCT	2 (0.0%)	2 (0.0%)	0 (0.0%)	1.00
SOT	3 (0.1%)	3 (0.1%)	0 (0.0%)	1.00
Corticosteroid use	233 (4.3%)	177 (4.2%)	56 (4.9%)	0.27
Antineoplastic agents	9 (0.2%)	7 (0.2%)	2 (0.2%)	1.00
Anti-CD20 therapy	1 (0.0%)	1 (0.0%)	0 (0.0%)	1.00
Prophylaxis for PJP	20 (0.4%)	14 (0.3%)	6 (0.5%)	0.33

Data are presented as median (interquartile range) for continuous variables and number (percentage) for categorical variables. P values were calculated using the Mann–Whitney U test for continuous variables and the chi-squared test or Fisher’s exact test for categorical variables.

Abbreviations: *COPD* chronic obstructive pulmonary disease; *HSCT* hematopoietic stem cell transplantation; *PJP* *Pneumocystis jirovecii* pneumonia; *SOT* solid organ transplantation.

### Household transmission to the partner

**Fig. 2:**
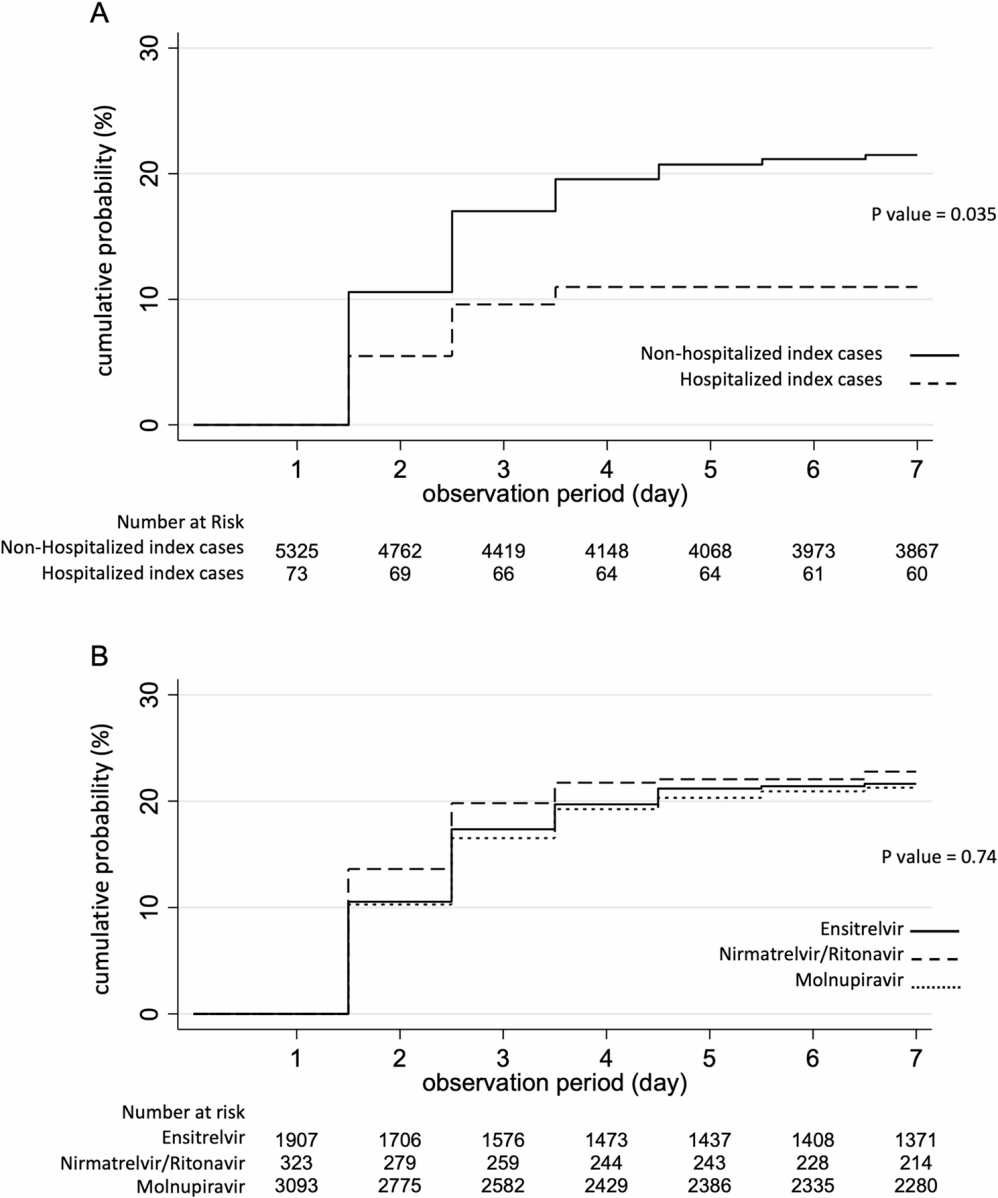
Cumulative probability of COVID-19 diagnosis in household partners Kaplan–Meier curves were used to estimate the 7-day cumulative probability of subsequent infection in the spouse after the start of the antiviral treatment for the index case. Day 0 is defined as the diagnosis date of the index patient A. Household transmission compared between index patients who were hospitalized on day 0 and those treated as outpatients B. Household transmission based on the antiviral agent administered to the index patient: ensitrelvir, nirmatrelvir/ritonavir, or molnupiravir Day 0 was defined for each household as the earliest day of COVID-19 diagnosis between the two household members P values were calculated using the log-rank test

Between day 2 and day 7, secondary transmission to the partner had occurred in 1,143 households (21.2%, 1,143/5,398). To evaluate the potential impact of isolation due to hospitalization, transmission rates were compared between hospitalized and non-hospitalized index patients (Fig. 2A). The cumulative transmission rate by day 7, estimated using the Kaplan–Meier method, was 11.0% (95% confidence interval [CI], 5.7–20.8%, *n* = 73) among hospitalized patients and 21.5% (95% CI, 20.4–22.6%, *n* = 5,325) among outpatients (log-rank test, *p* = 0.035). Among outpatients, the transmission rates by antiviral treatment were 21.3% (95% CI, 19.9–22.8, *n* = 3,093) for molnupiravir, 22.8% (95% CI, 18.6–27.8, *n* = 323) for nirmatrelvir/ritonavir, and 21.6% (95% CI, 19.8–23.6, *n* = 1,907) for ensitrelvir (log-rank test, *p* = 0.74).

### Cox proportional hazards analysis

Before modeling, the proportional hazards assumption was tested using Schoenfeld residuals. Variables such as multiple myeloma in index patients and dialysis in index patients violated the assumption and were excluded from the multivariable analysis.

The results of the Cox proportional hazards analysis are provided in Table 2. In the univariable analysis, male sex in the index case was significantly associated with an increased risk of household transmission, whereas a history of COVID-19 in the index patient and in the partner was associated with a reduced risk.

**Table 2:** Cox proportional hazards models for household transmission of COVID-19

		Univariable		Multivariable	
	Events/Total	HR (95% CI)	*P* value	HR (95% CI)	*P* value
**Treatment of Index patients**					
Outpatient molnupiravir	653/3093	Reference			
Outpatient ensitrelvir	409/1907	1.02 (0.90–1.16)	0.74	0.98 (0.86–1.12)	0.79
Outpatient nirmatrelvir/ritonavir	73/323	1.09 (0.86–1.39)	0.47	1.10 (0.86–1.40)	0.45
Outpatient remdesivir	0/2	NA	NA	NA	NA
Hospitalized and treated on day 0	8/73	0.50 (0.25–1.00)	0.052	0.51 (0.25–1.03)	0.062
**Index patient characteristics**					
Age					
<49 years	298/1029	Reference			
49–55 years	266/982	0.94 (0.80–1.11)	0.47	0.94 (0.79–1.11)	0.47
56–60 years	304/1162	0.92 (0.78–1.08)	0.28	0.88 (0.75–1.04)	0.14
≥61 years	275/1082	0.89 (0.76–1.05)	0.18	0.84 (0.71–1.00)	0.051
Sex	811/3444	1.43 (1.26–1.62)	< 0.001	1.43 (1.26–1.63)	< 0.001
Female	332/1954	Reference			
Oxygen use on day 0	1/24	0.18 (0.03–1.31)	0.09		
No	1142/5374	Reference			
History of COVID-19	23/253	0.40 (0.26–0.60)	< 0.001	0.50 (0.33–0.76)	0.001
No	1120/5145	Reference			
Solid tumor	117/544	1.02 (0.84–1.24)	0.82	1.06 (0.87–1.29)	0.56
No	1026/4854	Reference			
Leukemia	1/13	0.35 (0.05–2.52)	0.30	0.42 (0.06–3.05)	0.39
No	1142/5385	Reference			
Lymphoma	6/39	0.72 (0.32–1.61)	0.42	0.78 (0.35–1.76)	0.55
No	1137/5359	Reference			
Multiple myeloma	3/14	0.99 (0.32–3.09)	0.99	1.07 (0.34–3.34)	0.91
No	1140/5384	Reference			
Heart failure	89/391	1.10 (0.88–1.36)	0.40		
No	1054/5007	Reference			
Asthma	59/252	1.13 (0.87–1.47)	0.35		
No	1084/5146	Reference			
COPD	19/84	1.09 (0.69–1.72)	0.70		
No	1124/5314	Reference			
Interstitial lung disease	25/98	1.22 (0.82–1.82)	0.32		
No	1118/5300	Reference			
Liver cirrhosis	1/17	0.27 (0.04–1.91)	0.19		
No	1142/5381	Reference			
Dialysis	9/53	0.77 (0.40–1.49)	0.44		
No	1134/5345	Reference			
Diabetes mellitus	166/712	1.13 (0.96–1.33)	0.14		
No	977/4686	Reference			
Collagen disease	71/326	1.04 (0.82–1.32)	0.76		
No	1072/5072	Reference			
HSCT	0/1	Not included			
SOT	3/15	0.91 (0.29–2.83)	0.87		
No	1140/5383	Reference			
Corticosteroid use	56/305	0.85 (0.65–1.11)	0.23	0.95 (0.72–1.26)	0.74
No	1087/5093	Reference			
Antineoplastic agents	0/6	Not included			
Anti-CD20 therapy	0/6	Not included			
Prophylaxis for PJP	7/44	0.73 (0.35–1.54)	0.42	0.93 (0.42–2.04)	0.85
No	1136/5354	Reference			
**Partner characteristics**					
Age					
<48 years	287/1009	Reference			
48–54 years	255/931	0.97 (0.82–1.14)	0.68		
55–59 years	280/1032	0.96 (0.81–1.13)	0.60		
≥60 years	321/1283	0.89 (0.76–1.05)	0.16		
History of COVID-19	28/426	0.28 (0.19–0.40)	< 0.001	0.31 (0.21–0.45)	< 0.001
No	1115/4972	Reference			
Solid tumor	90/448	0.95 (0.76–1.17)	0.61		
No	1053/4950	Reference			
Leukemia	2/10	0.94 (0.24–3.78)	0.94		
No	1141/5388	Reference			
Lymphoma	5/25	0.94 (0.39–2.26)	0.89		
No	1138/5373	Reference			
Multiple myeloma	1/3	1.77 (0.25–12.54)	0.57		
No	1142/5395	Reference			
Heart failure	49/223	1.04 (0.78–1.39)	0.78		
No	1094/5175	Reference			
Asthma	41/216	0.89 (0.65–1.22)	0.48		
No	1102/5182	Reference			
COPD	5/40	0.57 (0.24–1.37)	0.21		
No	1138/5358	Reference			
Interstitial lung disease	19/72	1.27 (0.80–1.99)	0.31		
No	1124/5326	Reference			
Liver cirrhosis	4/19	1.01 (0.38–2.71)	0.98		
No	1139/5379	Reference			
Dialysis	3/10	1.56 (0.50–4.83)	0.45		
No	1140/5388	Reference			
Diabetes mellitus	93/422	1.05 (0.85–1.29)	0.67		
No	1050/4976	Reference			
Collagen disease	71/301	1.13 (0.89–1.44)	0.32		
No	1072/5097	Reference			
HSCT	0/2	Not included			
SOT	0/3	Not included			
Corticosteroid use	56/233	1.15 (0.88–1.50)	0.31		
No	1087/5165	Reference			
Antineoplastic agents	2/9	1.03 (0.26–4.11)	0.97		
No	1141/5389	Reference			
Anti-CD20 therapy	0/1	Not included			
Prophylaxis for PJP	6/20	1.41 (0.63–3.13)	0.41		
No	1137/5378	Reference			

Abbreviations: *CI* confidence interval; *COPD* chronic obstructive pulmonary disease; *HR* hazard ratio; *HSCT* hematopoietic stem cell transplantation; *NA* not applicable; *PJP* *Pneumocystis jirovecii* pneumonia; *SOT* solid organ transplantation.

The multivariable Cox model included antiviral treatment group, age, sex, prior COVID-19 infection, hospitalization, and immunocompromised conditions (e.g., solid tumors, leukemia, lymphoma, multiple myeloma, corticosteroid use, and prophylaxis for PJP), which were selected a priori based on clinical relevance. No additional variables showed statistically significant associations in the univariable analysis. In the multivariable analysis, male sex of the index case (adjusted HR, 1.43; 95% CI, 1.26–1.63; *p* < 0.001), a history of COVID-19 in the index patient (adjusted HR, 0.50; 95% CI, 0.33–0.76; *p* = 0.001), and a history of COVID-19 in the partner (adjusted HR, 0.31; 95% CI, 0.21–0.45; *p* < 0.001) remained significant after adjusting for possible risk factors. Although not statistically significant, hospitalization (i.e., hospitalized treatment mostly with remdesivir) tended to be associated with a lower risk of transmission (adjusted HR, 0.51; 95% CI, 0.25–1.03; *p* = 0.062). Index patients aged ≥ 61 years showed a trend toward a lower hazard of household transmission compared with those aged < 49 years (adjusted HR, 0.84; 95% CI, 0.71–1.00; *p* = 0.051).

### Sensitivity analysis

As a sensitivity analysis, we excluded 19 households in which the index patient was hospitalized between days 1 and 7, as well as two households in which the partner was hospitalized for reasons unrelated to COVID-19 during the same period. The cumulative transmission rate by day 7 was consistent with the main analysis (outpatient group: 21.5% [95% CI, 20.4–22.7]; inpatient group: 11.0% [5.7–20.8]; *p* = 0.034). Transmission rates among the three outpatient antiviral agents also remained similar (molnupiravir: 21.3% [19.9–22.8]; nirmatrelvir/ritonavir: 22.9% [18.7–28.0]; ensitrelvir: 21.7% [19.9–23.6]; *p* = 0.70). The results of the multivariable analysis were also largely unchanged (e.g., hospitalization: adjusted HR, 0.51; 95% CI, 0.25–1.03; *p* = 0.062; index patients aged ≥ 61 years: adjusted HR, 0.84; 95% CI, 0.71–1.00; *p* = 0.055).

## Discussion

In this study, we analyzed large administrative claims data to evaluate the impact of antiviral agents on the household transmission of SARS-CoV-2. To the best of our knowledge, this is the first study to assess whether the impact of different antiviral agents on household transmission varies. Importantly, this study was not designed to evaluate the efficacy of these agents in preventing disease progression, but rather focused on their potential effect in reducing secondary transmission within households. Although nirmatrelvir/ritonavir showed the strongest evidence for preventing severe disease and faster viral clearance than molnupiravir [25], household transmission rates were not statistically different among the three outpatient oral antiviral agents. Although not statistically significant, hospitalization of the index patients was associated with a trend toward lower incidence of subsequent infection of the spouse, likely reflecting the effect of physical isolation.

Theoretically, reducing viral loads should lead to a decrease or at least no increase in secondary transmission. However, this hypothesis is yet to be confirmed clinically for COVID-19. Reduced secondary transmission of SARS-CoV-2 has been demonstrated in an animal model [16]. However, in that study, uninfected animals were exposed to the index animals only after the infected animals received antiviral treatment. In contrast, in real-world settings, household members are typically in contact before the initiation of treatment, which may limit the effectiveness of antivirals in reducing the transmission of the virus. Although influenza antivirals have shown high efficacy in both post-exposure prophylaxis and reduction in household transmission [14, 15, 26], similar results have not been observed for SARS-CoV-2 [4, 27]. This discrepancy may be attributed to differences in the virological characteristics or mechanisms of antivirals, and SARS-CoV-2 can be shed before symptom onset [17], making early intervention more challenging. However, although the reduction in transmission among hospitalized patients in our study did not reach statistical significance (*p* = 0.062), it may still suggest that physical isolation after symptom onset may still help prevent secondary infections.

The comparative effects of different antivirals in reducing the risk of SARS-CoV-2 transmission have not been consistent in the literature. The aforementioned animal study has reported that molnupiravir was more effective than nirmatrelvir/ritonavir in preventing secondary transmission [16]. In contrast, a clinical trial showed that the viral clearance was faster after nirmatrelvir/ritonavir than after molnupiravir [25]. These contradictory results imply that the ability to reduce viral spread may not necessarily correlate with clinical efficacy or viral load reduction. Another possible reason is the higher viral rebound frequency after nirmatrelvir/ritonavir treatment than after molnupiravir treatment [25]. In our study, household transmission was slightly higher among patients treated with nirmatrelvir/ritonavir particularly between day1 and day 3, although the overall difference was not statistically different among the three outpatient oral antiviral agents. Although this weak finding was aligned with that of the animal study, it could also be due to the potential difference in the time from infection between the different treatments, which was not available in our data. In our study, the number of subsequent household infections peaked on day 2 in the nirmatrelvir/ritonavir group, whereas it decreased monotonously for the other two drugs (data not shown). Considering that the theoretical distribution of secondary infection is a bell-shaped curve, this may indicate that nirmatrelvir/ritonavir was administered slightly earlier in the disease course compared with other antivirals, which could explain the higher risk in the nirmatrelvir/ritonavir group in the first few days. At the same time, earlier administration of antiviral should be theoretically more effective in reducing the viral loads and subsequent risk of transmission. Further investigation is needed to determine whether the preventive effects differ among antiviral agents, accounting for the differential risks of secondary infections over the course of the disease.

Our study also explored the patient characteristics associated with the risk of household transmission. Notably, male index patients were more likely to transmit infection to their partners. Although previous studies on household transmission of COVID-19 have not reported significant sex-related differences [6, 7], a study using the same JMDC database for influenza reported that male index patients had higher secondary transmission rates [15]. In most households in our cohort, the husband was the primary insured individual, and the wife was a dependent spouse. When male index patients developed COVID-19, they may spend more time recovering at home, increasing the risk of transmission to their wives. Alternatively, differences in healthcare-seeking behaviors between males and females may have contributed to this [28]. A similar pattern was previously reported on antibiotic prescription: females received 16% more antibiotics in Japan [29]. In one previous study, older age in household contacts was reported to be associated with an increased risk of secondary transmission of SARS-CoV-2 [7]. In our study, however, older age in the index patients (and also in their spouses, which were highly correlated) was associated with a trend toward lower transmission. This may reflect greater adherence to infection control measures or higher vaccination rate among older adults in Japan [30]. We hypothesized that immunocompromised patients might have higher viral shedding and an increased risk of household transmission; however, we did not observe significant associations in this subgroup. This may reflect a greater awareness of infection control practices among immunocompromised individuals, similar to the situation in the elderly. A history of prior COVID-19 infection in either the index patient or the partner was associated with a reduced risk of transmission and/or disease progression, likely due to acquired immunity. Other comorbidities, such as cardiac and respiratory diseases, did not significantly influence household transmission. Although ensitrelvir, unlike other antivirals, can be prescribed to patients without underlying conditions, we did not observe any differences after adjustment for characteristics known to influence disease progression. Moreover, comorbidities were not associated with the risk of household transmission.

The incidence of subsequent COVID-19 diagnosis in the spouses was used as a surrogate for secondary household transmission. As a certain proportion of infected individuals could remain asymptomatic or do not seek medical care even after developing symptoms, it is likely that our data might have underestimated the true rate of secondary household transmission. Household transmission rates were reported to be approximately 30–50% in the Omicron era [4–7]. In our cohort, including those excluded due to diagnoses on day 0 or day 1, approximately 30% of households in which at least one member received antiviral treatment had both spouses diagnosed with COVID-19. Therefore, we think substantial underestimation was unlikely.

This study has certain limitations. First, the vaccination history was not available, and we assumed similar vaccination rates across the antiviral treatment groups. Vaccination rates may differ substantially between treated and untreated patients because this is a key factor in deciding the clinical management plan. We believe, however, that there were no major differences among the different the antiviral treatment groups. It is also possible that hospitalized patients (i.e., those with severe COVID-19) had lower vaccination rates, which can lead to higher transmission risk in this group than those treated at outpatient. This potential confounding could have underestimated our finding on the greater reduction of the risk in hospitalized patients, but overestimation is not likely.

Second, our database does not contain information on symptom onset. However, according to the prescribing information approved in Japan, all antiviral agents are recommended to be initiated soon after diagnosis, and we believe that the timing of administration did not substantially differ among agents. Notably, only ensitrelvir is explicitly indicated for use within 72 h of symptom onset based on clinical trial data, suggesting that it may have been prescribed earlier than other antivirals. Nevertheless, the Kaplan–Meier curves for transmission were closely aligned across treatment groups, suggesting that any differences in timing were unlikely to have had a major impact on the overall findings.

Third, we assumed that subsequent infections in the married partners under in the same health insurance represented secondary household transmission; however, simultaneous infections or different infection sources could not be ruled out. However, accurately identifying the transmission route is challenging even in clinical settings, not only in large database studies.

Fourth, family structures based on insurance records may not accurately reflect actual living arrangements, which could differ in some cases (e.g., multi-generational households, or temporary separations). Although these factors may have affected the estimated overall risk of household transmission, they were unlikely to have significantly influenced antiviral treatment decisions confounding our comparison results.

## Conclusions

This is the first study to evaluate the differences in household transmission of SARS-CoV-2 across different antiviral treatments. Household transmission rates were not statistically different among three different outpatient oral antiviral agents. Hospitalization was associated with a trend toward reduced secondary infections, suggesting that even after symptom onset, isolation may be effective in preventing transmission.

## Supplementary Information

Supplementary Material 1

## Data Availability

The data used in this study were obtained from JMDC Co., Ltd., and are not publicly available because of contractual and privacy restrictions. Relevant analytic data are available from the corresponding author upon reasonable request at kikeuchi@g.ecc.u-tokyo.ac.jp.

## Abbreviations

CI: Confidence interval
COPD: Chronic obstructive pulmonary disease
COVID-19: Coronavirus disease 2019
HR: Hazard ratio
HSCT: Hematopoietic stem cell transplantation
ICD-10: International Classification of Diseases, 10th Revision
IQR: Interquartile range
PJP: Pneumocystis jirovecii pneumonia
SOT: Solid organ transplantation
