# Supplementary material for: Comparative effectiveness of antiviral treatment on household transmission of SARS-CoV-2: a retrospective cohort study using administrative data
Source: BMC Infect Dis. 2025 Sep 29;25:1213. doi: 10.1186/s12879-025-11651-6 (PMC12482191; doi:10.1186/s12879-025-11651-6)
Supplement: Supplementary file 1 — Supplementary Material 1 [file 12879_2025_11651_MOESM1_ESM.docx]

Supplementary Table 1. ICD-10 codes for case definition

| Diagnosis | ICD 10 code and ATC code |
| --- | --- |
| Solid tumor | ICD10: C00-76 |
| Lymphoma | ICD10: C81-88 |
| Leukemia | ICD10: C91-95 |
| Multiple myeloma | ICD10: C90.0 |
| Heart failure | ICD10: I50 ACT: C03A, 07A, 09A,C,D,X |
| Ashma | ICD-10: J45, 46 |
| COPD | ICD10: J43 |
| Interstitial lung disease | ICD-10: J84.0,1,8, B221, J704, M051, M321, 330-332, 351 |
| Liver cirrhosis | ICD10: K746 |
| Diabetes mellitus | ICD10: E10-14 ACT: A10 |
| Collagen diseases | ICD10: M05, 06, 30-35, 45, 79 |
| Solid organ transplant | ICD10: Z94.0-94.7, 94.9 |
| Hematopoietic stem cell transplantation | ICD10: Z94.8 |
| Abbreviations: ATC, anatomical therapeutic chemical classification; COPD, chronic obstructive pulmonary disease; ICD-10, international classification of diseases 10th revision | |
